# The Effect of Far-Infrared Therapy on the Peritoneal Membrane Transport Characteristics of Uremic Patients Undergoing Peritoneal Dialysis: An Open-Prospective Proof-of-Concept Study

**DOI:** 10.3390/membranes11090669

**Published:** 2021-08-30

**Authors:** Ching-Po Li, Chyong-Mei Chen, Chia-Hao Chan, Szu-Yuan Li, Ming-Tsun Tsai, Chun-Fan Chen, Yung-Tai Chen, Tz-Heng Chen, Fan-Yu Chen, Ching-Han Yang, Yi-Hsin Chou, Tsung-Yueh Wang, Ann Charis Tan, Chih-Ching Lin

**Affiliations:** 1 School of Medicine, National Yang Ming Chiao Tung University, Hsinchu 300, Taiwan; s19701093@hotmail.com (C.-P.L.); syli@vghtpe.gov.tw (S.-Y.L.); mingtsun74@gmail.com (M.-T.T.); b8701004@gmail.com (C.-F.C.); ytchen0117@gmail.com (Y.-T.C.); s19401021@gmail.com (T.-H.C.); nono007tw@gmail.com (F.-Y.C.); shinyhan12@gmail.com (C.-H.Y.); maochiu2@yahoo.com.tw (Y.-H.C.); tywang10@vghtpe.gov.tw (T.-Y.W.); 2 Division of Nephrology, Department of Medicine, Taipei Veterans General Hospital, Taipei 112, Taiwan; box033@gmail.com; 3 Institute of Public Health, School of Medicine, National Yang Ming Chiao Tung University, Hsinchu 300, Taiwan; cmchen2@nycu.edu.tw; 4 Institute of Clinical Medicine, National Yang Ming Chiao Tung University, Hsinchu 300, Taiwan; 5 Division of Nephrology, Department of Internal Medicine, National Yang Ming Chiao Tung University Hospital, Yilan 260, Taiwan; 6 Division of Nephrology, Department of Internal Medicine, Taipei City Hospital Heping Fuyou Branch, Taipei 100, Taiwan; 7 Center for General Education, National Taipei University, Taipei 104, Taiwan; 8 Division of Nephrology, Department of Medicine, Taipei Veterans General Hospital Yuli Branch, Hualien 981, Taiwan

**Keywords:** end-stage renal disease, D/P creatinine ratio, far-infrared therapy, peritoneal dialysis, peritoneal membrane transport

## Abstract

Long-term peritoneal dialysis (PD) can lead to detrimental changes in peritoneal membrane function, which may be related to the accumulation of glucose degradation products. A previous study demonstrated that 6 months of far-infrared (FIR) therapy may decrease glucose degradation products in PD dialysate. Due to limited literature on this matter, this study aims to investigate the effect of FIR therapy on the peritoneal membrane transport characteristics of PD patients. Patients were grouped according to baseline peritoneal transport status: lower transporters (low and low-average) and higher transporters (high-average and high). Both groups underwent 40 min of FIR therapy twice daily for 1 year. In lower transporters, FIR therapy increased weekly dialysate creatinine clearance (6.91 L/wk/1.73 m^2^; *p* = 0.04) and D/P creatinine (0.05; *p* = 0.01). In higher transporters, FIR therapy decreased D/P creatinine (−0.05; *p* = 0.01) and increased D/D0 glucose (0.05; *p* = 0.006). Fifty percent of high transporter patients shifted to high-average status after FIR therapy. FIR therapy may decrease D/P creatinine for patients in the higher transporter group and cause high transporters to shift to high-average status, which suggests the potential of FIR therapy in improving peritoneal membrane function in PD patients.

## 1. Introduction

Peritoneal dialysis (PD) has been proven to be an alternate choice compared to hemodialysis as a type of maintenance renal replacement therapy for patients with end-stage renal disease (ESRD) [1]. Long-term PD can lead to structural membrane changes that are believed to contribute to changes in solute transport and loss of ultrafiltration [2]. Long-term exposure to high glucose concentrations in conventional PD solutions has been one of the contributors to changes in peritoneal membrane structure and function. Glucose degradation products (GDP) generated in glucose-containing PD solutions may lead to neoangiogenesis, fibrosis, inflammation, and eventual peritoneal failure in chronic PD patients [3,4]. Moreover, high glucose concentrations may cause metabolic complications such as hyperglycemia and dyslipidemia, which may then lead to further cardiovascular complications [5,6].

Far-infrared (FIR) therapy utilizes FIR radiation with a wavelength range of 5.6–1000 μm that is perceived as heat by receptors in the skin [7]. The skin surface temperature can steadily progress to approximately 38–39 °C during FIR therapy for 30–60 min [8]. When compared with conventional thermal therapy methods, FIR therapy has considerably less risk of side effects such as burn injury, infection, risk of vascular access failure, or prolonged bleeding from the previous venipuncture site. A study we conducted showed that FIR therapy resulted in increased access flow of arteriovenous fistula in hemodialysis patients both after a single dialysis session and after a 1-year treatment course due to thermal effect-induced vasodilation [9]. Previous studies have reported the non-thermal properties treatment using infrared radiation, such as (1) inhibiting endothelial inflammation via heme oxygenase-1 (HO-1) induction that may lead to preserving the patency of arteriovenous fistulas in hemodialysis patients (FIR therapy) [10], (2) inhibiting neointimal hyperplasia in rabbits by decreasing the proliferation of vascular smooth muscle cells, possibly reducing the frequency of vascular access stenosis (non-ablative infrared laser therapy) [11], and (3) improving endothelial function by decreasing urinary 8-epi-prostaglandin F2α levels (oxidative stress marker) in patients with coronary risk factors (sauna therapy), [12] and reducing serum advanced glycation end-products and diabetes-induced inflammatory markers in the vascular endothelium of diabetic mice (FIR therapy) [13].

The majority of the literature on FIR therapy for patients with renal diseases was focused on hemodialysis patients [9,10,14,15]. To date, only 2 published studies explored the effect of FIR therapy on PD patients. A case study showed that 3 months of FIR therapy alleviated a PD patient’s gastrointestinal symptoms due to encapsulating peritoneal sclerosis, a rare yet severe complication of PD, as well as improved his nutritional status and weight [16]. A recent study also showed that 6 months of FIR therapy may decrease dialysate GDPs in 31 ESRD patients on PD [17]. Due to limited literature, this study aims to investigate the long-term effect of FIR therapy on the peritoneal membrane transport characteristics of ESRD patients on PD.

## 2. Materials and Methods

### 2.1. Study Design

This study was conducted at the nephrology unit of Taipei Veterans General Hospital in Taipei, Taiwan. Prior to subject recruitment, the study was conducted according to the guidelines of the Declaration of Helsinki, and approved by the Institutional Review Board of Taipei Veterans General Hospital. Patients who met the following criteria were included in the study: (1) ESRD patients between 18 and 90 years old who have not received any FIR therapy within the previous 12 months and (2) patients who have been on a standard continuous ambulatory PD (1.5–2 L; 4–5 exchanges/day) or automated PD program for more than 3 months. Patients with a history of PD-related peritonitis, cerebrovascular accident, myocardial infarction, and/or underwent interventional procedures (percutaneous transluminal coronary angioplasty or stent) for coronary artery disease within 3 months before the study period were excluded from the study.

There were 66 patients enrolled in the study. Written informed consent was obtained from all the patients involved. Patients were then allocated to 2 groups according to their baseline D/P creatinine ratio (peritoneal transport status groups according to Twardowski et al.), namely patients with lower transport characteristics (low: < 0.50 and low-average: 0.50 to 0.64) and patients with higher transport characteristics (high: ≥ 0.81 and high-average: 0.65 to 0.80) and both groups underwent FIR therapy for 1 year. The demographic and clinical data of the patients were recorded from the hospital’s database. The clinical data were recorded at 2 time points, namely pre-FIR therapy (at enrollment) and post-FIR therapy (at 1 year).

The demographic data consisted of the patients’ age, gender, weight (kg), PD duration (months), comorbidities, and medications. The clinical data included peritoneal function and serum biochemical parameters. The peritoneal function parameters consisted of Kt/V urea (dialysate, renal, and total), weekly creatinine clearance (CCr) (dialysate, renal, and total), normalized protein catabolic rate (nPCR, g/kg/day), D/D0 glucose (ratio of dialysate glucose after time of dwell to initial dialysate glucose), D/P creatinine (dialysate/plasma creatinine ratio at 4 h), D/P urea (dialysate/plasma urea ratio at 4 h), and ultrafiltration volume (mL). The serum biochemical parameters consisted of albumin (g/dL), fasting blood glucose (mg/dL), cholesterol (mg/dL), triglycerides (mg/dL), blood urea nitrogen (mg/dL), creatinine (mg/dL), sodium (mmol/L), potassium (mmol/L), calcium (mg/dL), and phosphate (mg/dL).

### 2.2. Far-Infrared (FIR) Therapy

**Figure 1:**
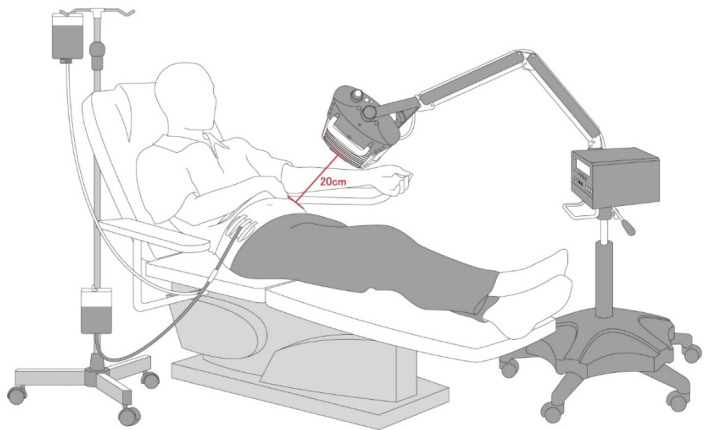
FIR therapy set-up during a PD exchange. Abbreviations: FIR: far-infrared; PD: peritoneal dialysis.

The WS TY101 FIR emitter (WS Far Infrared Medical Technology Co., Ltd., Taipei, Taiwan) was used to conduct the FIR therapy in this study. The electrified ceramic plates of the emitter can generate electromagnetic waves within the 3–25 μm range. The irradiating power density was set at 20 mW/cm^2^ and the top radiator was set at 20 cm above the abdomen (Figure 1). The patients underwent FIR therapy for 40 min twice daily during the draining and filling time of the first and last exchange of each PD session for 1 year.

### 2.3. Statistical Analysis

The study data were analyzed using IBM SPSS Statistics version 24. Continuous data were presented as mean and standard deviation and analyzed using an independent-samples t-test, while categorical data were presented as number and percentage and analyzed using a chi-square test. P values < 0.05 were considered statistically significant.

## 3. Results

### 3.1. Baseline Parameters of Patients

Table 1 shows the baseline parameters of the patients enrolled in the study where 62.12% of the patients were classified in the higher transporter group. Patients in the higher transporter group exhibited a significantly longer PD duration (44.68 vs. 20.16 months; *p* = 0.03). Age, sex, comorbidity, medication, physical examination, PD type, and PD prescription parameters between groups did not reach statistical significance.

During the study period, 22 patients dropped out from PD, due to a transition to hemodialysis (9 patients), kidney transplant (2 patients), hospital transfer (1 patient), and death (10 patients).

### 3.2. Effect of FIR Therapy on Peritoneal Membrane Function and Serum Biochemical Parameters

Table 2 shows the effect of FIR therapy on peritoneal membrane function and serum biochemical parameters of 66 PD patients after 1 year. Patients in the lower transporter group experienced a statistically significant increase in the weekly dialysate CCr (6.91 L/wk/1.73 m^2^; *p* = 0.04) and D/P creatinine ratio (0.05 units; *p* = 0.01), while patients in the higher transporter group experienced a significant decrease in the D/P creatinine ratio (−0.05 units; *p* = 0.01) and increase in the D/D0 glucose ratio (0.05 units; *p* = 0.006). It is therefore notable that there is a significant change in the D/P creatinine ratio between the lower transporter group (low and low-average) and higher transporter group (high and high-average) and that FIR therapy was beneficial in increasing the D/D0 glucose ratio while decreasing the D/P creatinine ratio in patients with higher transport characteristics.

A separate statistical analysis was performed on the 44-patient subgroup, which excluded patients who have dropped out from PD during the study period. Similar to the results of the 66-patient group, patients in the lower transporter group experienced a statistically significant increase in the D/P creatinine ratio (0.04 units; *p* = 0.045), while patients in the higher transporter group experienced a significant decrease in the D/P creatinine ratio (−0.05 units; *p* = 0.016) and increase in the D/D0 glucose ratio (0.05 units; *p* = 0. This subgroup analysis confirmed the effectiveness of FIR therapy on PD patients regardless of the sample size.

### 3.3. Effect of FIR Therapy on Peritoneal Transport Status

**Figure 2:**
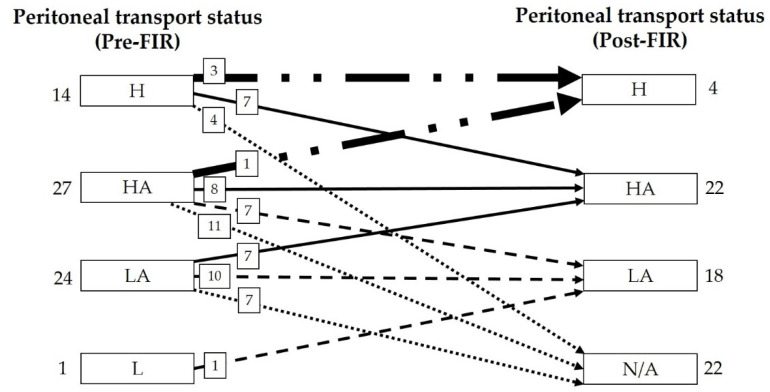
One-year effect of FIR therapy on peritoneal membrane transport status. Abbreviations: FIR: far-infrared; H: high; HA; high-average; LA: low-average; L, low; N/A: not available (patients dropped out during the study due to various reasons such as transition to hemodialysis, kidney transplant, hospital transfer, and death, as described in the first subsection of the results section).

Figure 2 shows the change in the number of patients of different peritoneal membrane transport status groups before and after 1 year of FIR therapy. Among 66 patients, there were 14 high transporters and 27 high-average transporters at the start of the study. After FIR therapy, 7 high transporters shifted to high-average transport status and 7 high-average transporters shifted to low-average transport status, indicating a decrease in the D/P creatinine ratio. Only 1 patient was considered as a low transporter at the start of the study and that same patient progressed to the low-average transport status after FIR therapy.

## 4. Discussion

The study explored the effects of FIR therapy on the peritoneal function of PD patients by assessing their peritoneal membrane transport characteristics. The main finding was that FIR therapy increased the weekly dialysate CCr and D/P creatinine ratio in the lower transporter group and decreased the D/P creatinine ratio for patients in the higher transporter group. It was also observed that 50% of high transporter patients shifted to high-average transport status after FIR therapy. These observed trends show that FIR therapy may be beneficial in improving peritoneal transport and peritoneal membrane function since previous studies like the CANUSA study have demonstrated that higher peritoneal transport was associated with decreased patient and technique survival in continuous ambulatory PD patients [18,19]. In a study on the longitudinal membrane function in functionally anuric patients treated with ambulatory PD, Davies et al. showed that after 12 months there was a significant 0.05 increase in the D/P creatinine ratio of all the enrolled patients in the study (from 0.75 to 0.8) and a significant 0.1 increase in the D/P creatinine ratio of the patient subgroup using glucose-only solutions (from 0.73 to 0.83) [20]. However, in this study, there was a significant 0.05 decrease in the D/P creatinine ratio in the high transporter group after 1 year of FIR therapy, which highlights the positive effect of FIR therapy on peritoneal transport. When compared with the values found in the study by Davies et al. serving as the control group, a net effect of 0.1 decrease in the D/P creatinine ratio can be estimated in patients who underwent 1 year of FIR therapy.

The exact therapeutic mechanisms of FIR therapy on the improvement of peritoneal function remain unknown. However, Masuda et al. reported that repeated sauna therapy may improve endothelial function and reduce oxidative stress, which in turn may protect against the progression and complications of coronary artery disease [12]. We have also conducted studies on hemodialysis patients where FIR therapy has clinically improved the flow, maturation, and patency of arteriovenous fistulas by inhibiting endothelial inflammation through the induction of HO-1 gene expression [9,10,15,21].

Previous in vitro studies showed that FIR therapy can improve endothelial cell function and exhibit anti-inflammatory properties [10,22]. Huang et al. showed in an animal study that FIR therapy may promote angiogenesis on local endothelial cells and restore endothelial progenitor cell function suppressed in a high-glucose environment [23]. There were also studies that demonstrated the microcirculation effects of FIR and thermal therapy on patients with diabetic foot and lymphedema, aiding in wound healing and microcirculation improvement. Cheng et al. showed that FIR radiation would improve blood circulation with evidence of surface temperature and blood flow image improvement after 3 months of treatment [24]. Liu et al. treated patients with lymphedema and lymphedematous skin by microwave and hot water immersion hyperthermia and showed near resolution of perivascular cellular infiltration, lymphatic lakes, and dilatation of blood capillaries [25].

**Figure 3:**
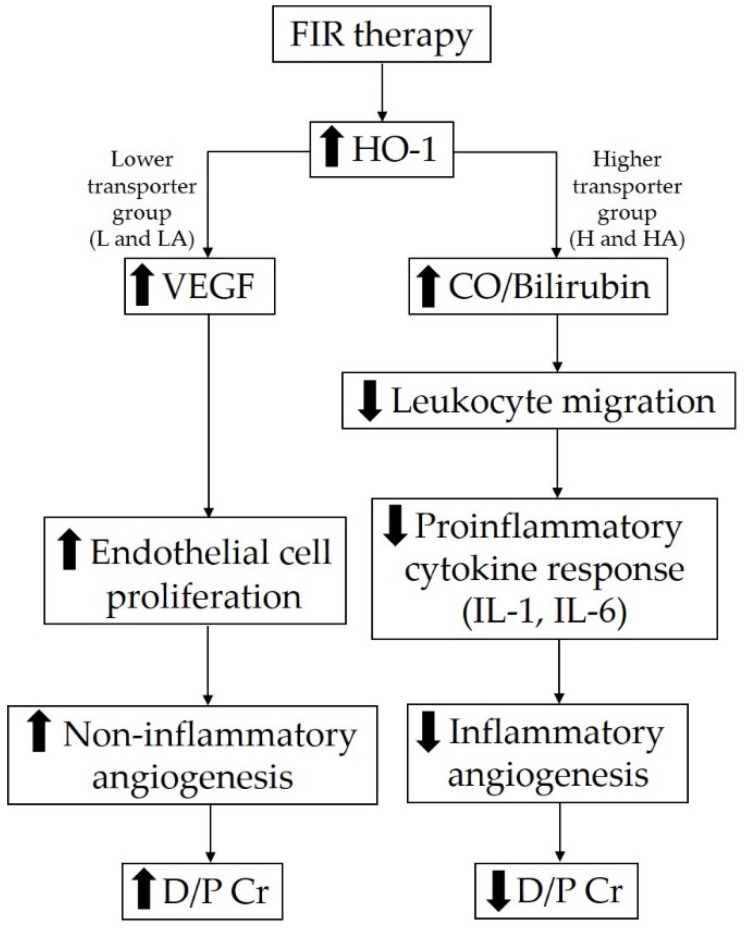
Probable mechanism of the effect of FIR therapy on angiogenesis via HO-1 induction. HO-1 activation by FIR therapy can increase vascular endothelial growth factor synthesis, inducing endothelial cell proliferation, thus stimulating non-inflammatory angiogenesis and increasing the D/P creatinine ratio in patients with lower peritoneal transport status. On the other hand, HO-1 breaks down heme into carbon monoxide and bilirubin (reduced from initial product biliverdin), which can inhibit leukocyte migration and proinflammatory cytokine release, thus suppressing inflammatory angiogenesis and decreasing the D/P creatinine ratio in patients with higher peritoneal transport status. Abbreviations: FIR, far-infrared; L, low; LA, low-average; H, high; HA, high-average; HO-1, heme oxygenase-1; VEGF, vascular endothelial growth factor; CO, carbon monoxide; IL-1, interleukin-1; IL-6, interleukin-6; D/P Cr, D/P, dialysate-to-plasma creatinine ratio.

When HO-1 is induced through FIR therapy, it increases the synthesis of vascular endothelial growth factor in endothelial cells, inducing endothelial cell proliferation, thus stimulating non-inflammatory angiogenesis. This may be why the D/P creatinine ratio increased in patients with lower transport characteristics [26,27,28]. The increased effective peritoneal capillary surface area due to angiogenesis stimulation through FIR therapy may also possibly increase the D/P creatinine ratio in patients in the lower transporter group [29]. Meanwhile, HO-1 is also a catalyst that breaks down heme into iron, carbon monoxide, and biliverdin, which is then subsequently reduced to bilirubin. Carbon monoxide and bilirubin can inhibit leukocyte migration and proinflammatory cytokine release, thus suppressing inflammatory angiogenesis. This may be why the D/P creatinine ratio decreased in patients with higher transport characteristics after FIR therapy [26,27,28]. Patients in the higher transporter group also had a significantly longer PD duration. Cho et al. showed that the use of conventional PD solutions and longer PD duration are two possible causes of inflammation in PD patients [30]. Patients in the higher transporter group may initially be in a more severe state of inflammation than those in the lower transporter group. The probable mechanism of the effect of FIR therapy on angiogenesis via HO-1 pathways is illustrated in Figure 3.

Chang et al. demonstrated that after the patients underwent 6 months of FIR therapy, 3 GDPs decreased significantly, namely methylglyoxal, furfural, and 5-hydroxymethyl-2-furaldehyde. Formaldehyde also exhibited a decreasing trend that approached borderline significance [17]. Previous studies have demonstrated that high levels of GDPs were linked to cellular damage, chronic inflammation, and oxidative stress [31]. Accumulation of aldehydes can also damage membrane lipids, cellular proteins, mitochondrial function, RNA and DNA and disrupt cell signaling [32]. This study is the first in vivo human study that demonstrated the effect of FIR therapy in decreasing dialysate GDP concentrations, which may be a possible reason why FIR therapy was also able to improve the peritoneal transport status of patients in the higher transporter group in this study.

Ou et al. showed in a previous case study that FIR therapy improved the gastrointestinal symptoms due to encapsulating peritoneal sclerosis, a rare yet severe complication of PD, of a patient who had received 13 years of continuous ambulatory PD, which may also be related to the anti-inflammatory action of the HO-1 pathway. After 3 months of FIR therapy, his bowel movement improved, he was able to tolerate a regular diet orally, and the ascites fluid became a light pink color after being initially dark red upon admission. The follow-up contrast-enhanced computed tomography scan showed an improvement in the dilated loops of the small bowel. After 12 months of FIR therapy, the patient’s ascites fluid became even lighter in color and there was further improvement in the nutritional status with a significant increase in the serum albumin level from 2.7 g/dL to 4.2 g/dL and body weight from 59.1 kg to 62.9 kg [10,16].

There are several limitations of the study that must be acknowledged. First, this was a single-center pilot study with a relatively small number of patients and an absence of a control group for comparison. Future large-scale randomized controlled trials are needed to confirm the preliminary results of this study. Second, a short follow-up period (1 year) was observed. Possible long-term effects of FIR therapy should be explored accordingly. Third, evidence of the effect of FIR therapy on the peritoneal microcirculation in PD patients is still lacking. Thus, future clinical studies are required to confirm the effects of FIR on human mesothelial and endothelial functions, as well as the proposed regulatory mechanism of FIR therapy on the alterations of the peritoneal membrane structure and function of PD patients.

To the best of our knowledge, this is the first study conducted that evaluated the effects of FIR therapy on the peritoneal membrane function of PD patients. Since FIR therapy is conducted during the first exchange (morning time) and last exchange (nighttime) of the PD session, it did not interfere with the patients’ daily activities and work schedule, reducing the inconvenience it may cause. Therefore, FIR therapy is convenient, non-invasive, relatively low-cost, and low-threshold, which makes it a promising treatment modality to improve peritoneal membrane transport characteristics in ESRD patients on long-term PD.

## 5. Conclusions

The results demonstrate that 1 year of FIR therapy decreased the D/P creatinine ratio for patients in the higher transporter group and caused high transporters to shift to high-average status, which suggests that FIR therapy is a potential treatment modality that may be able to improve peritoneal membrane function in PD patients.

## Figures and Tables

**Table 1:** Comparison of baseline parameters of patients grouped according to baseline peritoneal transport status.

Parameters	Peritoneal Transport Status		
	Lower (L and LA) n = 25 (37.88%)	Higher (H and HA) n = 41 (62.12%)	*p*
Age, years	59.72 ± 10.50	58.44 ± 17.01	0.71
**Sex**			
Male	11 (44%)	18 (43.90%)	0.99
Female	14 (56%)	23 (56.10%)	
**Comorbidities**			
Diabetes mellitus	10 (40%)	16 (39.02%)	0.94
Diabetic nephropathy	7 (28%)	15 (36.59%)	0.47
Hypertension	23 (92%)	37 (90.24%)	0.81
Hyperlipidemia	14 (56%)	15 (36.59%)	0.12
CAD/MI	3 (12%)	11 (26.83%)	0.15
Stroke	2 (8%)	2 (4.88%)	0.61
CHF	7 (28%)	5 (12.20%)	0.11
Gout	12 (48%)	15 (36.59%)	0.36
**Medications**			
Diuretics	5 (20%)	11 (26.83%)	0.53
Statins	15 (60%)	21 (51.22%)	0.49
Fibrates	2 (8%)	0 (0%)	0.07
ARB	13 (52%)	23 (56.10%)	0.75
CCB	17 (68%)	25 (60.98%)	0.57
Nitrites	4 (16%)	4 (9.76%)	0.45
Apresoline	0 (0%)	1 (2.44%)	0.43
Alpha blockers	4 (16%)	9 (21.95%)	0.56
Beta blockers	10 (40%)	22 (53.66%)	0.28
**Physical Examination**			
Weight, kg	59.20 ± 12.30	62.71 ± 13.37	0.29
Systolic BP, mmHg	138.76 ± 14.28	141.83 ± 20.91	0.52
Diastolic BP, mmHg	77.68 ± 10.49	79.41 ± 14.60	0.61
**PD Type**			
CAPD	21 (84%)	32 (78.05%)	0.56
APD	4 (16%)	9 (21.95%)	
PD duration, months	20.16 ± 16.79	44.68 ± 66.32	0.03
**PD Prescription**			
1.36% glucose, L	4.58 ± 2.98	3.78 ± 3.12	0.31
2.3% glucose, L	2.98 ± 2.52	3.62 ± 3.04	0.75
3.86% glucose, L	0	0.04 ± 0.32	0.44
Glucose load, g/day	143.20 ± 44.04	139.16 ± 46.90	0.73
1.1% amino acid, L	2 (8%)	5 (12.20%)	0.59
7.5% icodextrin, L	11 (44%)	24 (58.54%)	0.25

Age, weight, physical examination, PD duration, and PD solution parameters were expressed as mean ± standard deviation. Sex, comorbidity, medication, and PD type parameters were expressed as number and percentage. † Ultrafiltration volume = outflow volume − inflow volume. Abbreviations: L, low; LA, low-average; H, high; HA, high-average; CAD, coronary artery disease; MI, myocardial infarction; CHF, congestive heart failure; ARB, angiotensin II receptor blockers; CCB, calcium channel blockers; BP, blood pressure; PD, peritoneal dialysis; CAPD, continuous ambulatory peritoneal dialysis; APD, ambulatory peritoneal dialysis.

**Table 2:** Effects of FIR therapy on peritoneal membrane function and serum biochemical parameters of patients.

Parameters	Peritoneal Transport Status					
	Lower (L and LA)		*p*	Higher (H and HA)		*p*
	Pre-FIR	Post-FIR		Pre-FIR	Post-FIR	
Weight, kg	59.18 ± 12.29	59.82 ± 13.04	0.87	62.71 ± 13.37	63.41 ± 15.29	0.84
**Peritoneal membrane function**						
Dialysate Kt/V	1.67 ± 0.38	1.79 ± 0.33	0.29	1.74 ± 0.43	1.85 ± 0.45	0.32
Renal Kt/V	0.27 ± 0.38	0.16 ± 0.19	0.28	0.28 ± 0.34	0.20 ± 0.36	0.37
Total * Kt/V	1.94 ± 0.33	1.95 ± 0.24	0.89	2.02 ± 0.34	2.05 ± 0.47	0.76
Weekly dialysate CCr, L/wk/1.73 m^2^	35.46 ± 11.24	42.37 ± 9.95	0.04	45.09 ± 10.64	44.24 ± 11.25	0.75
Weekly renal CCr, L/wk/1.73 m^2^	15.84 ± 26.41	9.38 ± 13.30	0.35	14.33 ± 17.11	10.50 ± 17.22	0.37
Weekly total * CCr, L/wk/1.73 m^2^	51.30 ± 24.62	51.75 ± 14.51	0.86	59.42 ± 14.24	54.74 ± 16.44	0.24
NPCR	1.06 ± 0.24	1.11 ± 0.23	0.47	1.09 ± 0.27	1.08 ± 0.30	0.96
D/D0 glucose	0.44 ± 0.04	0.42 ± 0.04	0.17	0.31 ± 0.07	0.36 ± 0.07	0.006
D/P Cr	0.58 ± 0.04	0.63 ± 0.07	0.01	0.76 ± 0.08	0.71 ± 0.09	0.01
D/P urea	0.88 ± 0.10	0.85 ± 0.10	0.32	0.95 ± 0.11	0.92 ± 0.18	0.46
Ultrafiltration volume, mL	913.24 ± 495.67	958.22 ± 443.75	0.76	867.68 ± 499.92	926.96 ± 571.44	0.65
**Serum biochemistry**						
Albumin, g/dL	3.66 ± 0.39	3.62 ± 0.41	0.72	3.39 ± 0.45	3.39 ± 0.44	0.94
Glucose, mg/dL	108.24 ± 18.58	119.76 ± 46.92	0.27	128.88 ± 66.28	135.61 ± 70.59	0.68
Cholesterol, mg/dL	172.16 ± 33.76	170.48 ± 36.04	0.87	169.80 ± 36.68	175.23 ± 42.07	0.57
Triglycerides, mg/dL	146.24 ± 60.36	152 ± 95.56	0.81	130.34 ± 78.47	135.70 ± 84.25	0.78
BUN, mg/dL	73.08 ± 19.06	72.77 ± 19.08	0.96	75.10 ± 18.36	70.31 ± 24.43	0.34
Cr, mg/dL	11.83 ± 3.07	11.84 ± 3.17	0.99	11.20 ± 3.03	10.94 ± 3.73	0.74
Na, mmol/L	135.44 ± 4.30	134.82 ± 4.03	0.61	135.10 ± 3.97	135.97 ± 4.09	0.37
K, mmol/L	4.21 ± 0.79	4.06 ± 0.58	0.47	4 ± 0.56	3.88 ± 0.81	0.48
Ca, mg/dL	9.36 ± 0.73	9.33 ± 0.63	0.87	9.20 ± 0.79	9.39 ± 0.76	0.30
P, mg/dL	5.68 ± 1.53	5.47 ± 1.27	0.63	4.83 ± 1.30	5.29 ± 1.32	0.16

* Total = dialysate + renal. Abbreviations: L, low; LA, low-average; H, high; HA, high-average; PD, peritoneal dialysis; CCr, creatinine clearance; D/P Cr, dialysate/plasma creatinine ratio at 4 h; nPCR, normalized protein catabolic rate; D/D0 glucose, dialysate glucose-to-initial dialysate ratio; BUN, blood urea nitrogen; Cr, creatinine; Na, sodium; K, potassium; Ca, calcium; P, phosphate.

## Data Availability

Data sharing not applicable.
